# Plant Cells under Attack: Unconventional Endomembrane Trafficking during Plant Defense

**DOI:** 10.3390/plants9030389

**Published:** 2020-03-21

**Authors:** Guillermo Ruano, David Scheuring

**Affiliations:** 1 School of Plant Sciences and Food Security, Tel Aviv University, Tel Aviv 6997801, Israel; 2 Plant Pathology, University of Kaiserslautern, 67633 Kaiserslautern, Germany

**Keywords:** plant endomembrane system, plant pathogens, plant defense, vacuole, ER bodies, exocytosis

## Abstract

Since plants lack specialized immune cells, each cell has to defend itself independently against a plethora of different pathogens. Therefore, successful plant defense strongly relies on precise and efficient regulation of intracellular processes in every single cell. Smooth trafficking within the plant endomembrane is a prerequisite for a diverse set of immune responses. Pathogen recognition, signaling into the nucleus, cell wall enforcement, secretion of antimicrobial proteins and compounds, as well as generation of reactive oxygen species, all heavily depend on vesicle transport. In contrast, pathogens have developed a variety of different means to manipulate vesicle trafficking to prevent detection or to inhibit specific plant responses. Intriguingly, the plant endomembrane system exhibits remarkable plasticity upon pathogen attack. Unconventional trafficking pathways such as the formation of endoplasmic reticulum (ER) bodies or fusion of the vacuole with the plasma membrane are initiated and enforced as the counteraction. Here, we review the recent findings on unconventional and defense-induced trafficking pathways as the plant´s measures in response to pathogen attack. In addition, we describe the endomembrane system manipulations by different pathogens, with a focus on tethering and fusion events during vesicle trafficking.

## 1. Introduction

During the evolution of multicellular organisms, close relationships between plants and a plethora of different microorganisms have been established. Interactions can be either beneficial (symbionts) or harmful (pathogens) for the host. In the case of pathogenic interactions, plant immune responses, which differ dramatically from that of mammals, are initiated. Plants lack an adaptive immune system, characterized by specialized cells and antibodies, but survive constant and highly diverse attacks from numerous pathogens nevertheless. 

If pathogens can overcome preformed plant barriers (cuticle, cell wall) or even be sensed before [1], the so-called first line of defense is activated. Here, Pattern Recognition Receptors (PRRs) at the plasma membrane (PM) recognize conserved pathogen or microbial signatures (Pathogen-Associated Molecular Patterns (PAMPs)). In addition, PRRs can recognize self-wounding elements produced by the action of the pathogens (DAMPs, Damage-Associated Molecular Patterns). Receptor-ligand binding leads to PAMP-triggered immunity (PTI), which involves transcriptional changes in the nucleus as well as the induction of a set of instant immune responses. The latter include cell-wall fortification by callose deposition, calcium influx, stomatal closure and the production of reactive oxygen species (ROS) to create highly anti-microbial conditions [2]. For successful plant invasion, pathogens must not only penetrate preformed barriers but also have to bypass or suppress PTI. Therefore, many pathogens secrete proteinaceous effector molecules, which perturb intracellular processes [3] and thus downregulate PTI signaling and interfere with host plant defense. Some effectors are secreted in the plant apoplast, others into the cytosol [4].

To counteract effector-mediated suppression of PTI, plants have evolved a second line of defense, the so-called effector-triggered immunity (ETI). Here, effectors are recognized mostly in the cytosol by plant receptors known as R proteins, but also in the apoplast. Many of the R proteins share common structural features with Nucleotide-binding site Leucine-rich Repeat proteins (NLRs). In the past, receptor binding and subsequent induction of ETI as defense response led to the term avirulence (Avr) factors for many secreted effectors. In general, ETI is a potentiation of PAMP-triggered immune responses and often leads to a local hypersensitive response (HR) that causes programmed cell death (PCD) at the infection site to interfere with further pathogen proliferation. This initial local response later promotes an enhanced defensive state to peripheral cells and the whole plant in a process known as System Acquired Resistance (SAR) [5]. Although a clear distinction between PTI and ETI is difficult [6], the amplitude of the response is always higher during ETI.

According to their source of nutrients, plant pathogens can be classified into three groups: biotrophic, hemibiotrophic and necrotrophic pathogens. While biotrophs require living host tissue as a source of nutrients, necrotrophs kill their host cells rapidly and feed on dead plant tissue. Hemibiotrophs possess an initial biotrophic phase, switching to a necrotrophic stage after a certain period of time [7]. These differences in lifestyle are reflected on several levels: biotrophs produce and secrete an array of different effectors to suppress PTI and the amplitude of plant responses is rather low upon detection. Necrotrophs on the other hand, often secrete toxic cocktails of proteins, secondary metabolites and acids to overcome immune responses and to kill host plants quickly. The switch to necrotrophy of hemibiotrophic fungi is accompanied by a complete transcriptional reprogramming of gene expression. Initially, gene expression involves many secreted effectors while later many genes encoding virulence factors typical for necrotrophs are expressed [8,9,10,11].

**Figure 1:**
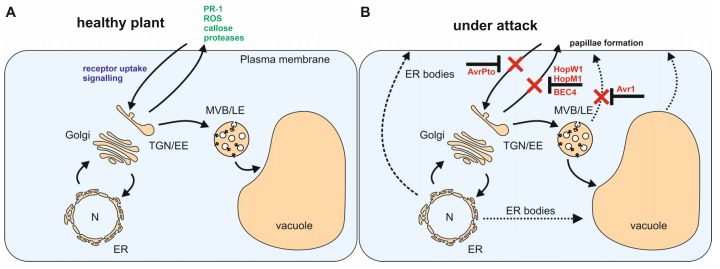
Pathogen-induced dynamics within the plant endomembrane system. (**A**) Different proteins and metabolites relevant for plant defense (green) can be secreted into the apoplast via the secretory pathway. Pathogen detection and signaling depends on the endocytosis of pattern recognition receptors (blue). (**B**) Pathogens have evolved effectors (red) to efficiently inhibit conventional trafficking routes and thus, functional plant defense. Upon pathogen attack, plants use unconventional trafficking pathways (dashed arrows) such as multivesicular bodies (MVB)/ late endosomes (LE) fusion with the plasma membrane (PM) and endoplasmic reticulum (ER) body formation for compensation.

Recently, increasing evidence indicates that dynamics within the endomembrane system are of fundamental importance for both - the first and the second line of defense. The plant endomembrane system is a complex intracellular membrane system, controlling the secretion of biomolecules and mediates the uptake of substances from the cell´s exterior and the delivery to specific intracellular locations. Compartments of the endomembrane system are connected via vesicle transport, but can also change composition, mature over time and migrate within a cell [12]. Targeted vesicle transport requires budding, tethering and fusion events at specific donor and target membranes respectively. The secretory pathway consists of the nuclear envelope, the endoplasmic reticulum (ER), the Golgi apparatus, endosomes, the vacuole and the PM (Figure 1A). Canonical plant trafficking routes include outward and vacuolar transport (secretory pathway) and inward transport (endocytic pathway) starting at the PM. Since there are already excellent reviews about PAMP-PRR recognition and downstream changes at the PM [13,14,15] we will focus in this review mainly on intracellular, effector-mediated changes during plant–pathogen interaction. We have organized the present article in individual sections, each covering a different plant cell organelle.

## 2. Endoplasmic Reticulum 

Secreted soluble proteins usually contain a signal peptide, which allows cotranslational translocation into the ER. From here, the secretory pathway leads via the Golgi apparatus and the trans-Golgi network (TGN) towards the PM and the apoplast as well as to the vacuole. Prior to protein export from the ER, correct folding and glycosylation has to be tightly controlled by the ER quality control system (ER-QC). Notably, individual PRRs are controlled with different stringencies. Functionality of the Elongation Factor Receptor (EFR, recognizing EF-tu bacterial peptide) depends on at least three *ELFIN* genes that encode potential components of ER-QC. In contrast, functionality of the flg22-sensitive 2 receptor (FLS2, recognizing a highly conserved 22-amino acid sequence of bacterial flagellin) is not affected upon *elfin* mutations. It was speculated that EFR, being only present in *Brassicaceae* may have evolved more recently than FLS2. Thus, correct folding and activity of FLS2 may depend less strongly on ER-QC [16]. Similarly, vacuolar sorting receptors (VSRs) responsible for targeting defense-related soluble proteins to the vacuole depend on N-linked glycosylation at the ER-Golgi interface to fulfill its function [17].

In addition to the ER-QC of membrane proteins, ER-derived vesicles (known as ER-bodies) accumulate defensive proteins such as pathogenesis-related 1 (PR1) or plant defensin 1.2 (PDF1.2) and different secondary metabolites involved in defense [18]. During the pathogen attack, levels of salicylic acid (SA) are increased and ER bodies increase in number and fuse with the PM to release their content (Figure 1B). However, not all ER bodies are able to fuse with the PM. Although PR1 and PR2 both contain a signal peptide for cotranslational translocation, only PR1-containing ER bodies are capable of fusion. In contrast, PR2 containing ER bodies remain in the cytoplasm [19]. In addition to this differential trafficking mechanism for both PRs, PR1 has been reported to follow the standard Golgi-dependent secretory route to the apoplast [20,21]. Notably, PR1 release into the apoplast is inhibited by the pathogenic fungus *Golovinomyces cichoracearum*. Upon infection, the pathogen specifically degrades the KEEP ON GOING (KEG) protein, which is involved in the secretion of apoplastic defense proteins. This leads to the misrouting of PR1 into the vacuole, potentially as part of the virulence strategy [22].

Another layer of defense has been added recently by the isolation of inducible ER bodies in Arabidopsis that accumulate atypical myrosinases in leaf cells during insect attack [23]. In general, myrosinases are responsible for hydrolyzing glucosinolates, thereby generating antimicrobial secondary metabolites such as thiocyanates, isothiocyanates and nitriles. Besides their presence in ER bodies, myrosinases are mainly stored in the vacuole, constituting the mustard oil bomb system of the plant. Under non-infected conditions, enzyme and substrate are stored in different compartments to exclude reaction and the entire system is mostly found in specialized idioblasts (myrosinase cells) close to phloem parenchyma [24]. Only upon cell disruption by feeding herbivores (e.g., aphids) both components come into contact and antimicrobial secondary metabolites are generated.

In addition to myrosinases, so-called vacuolar processing enzymes (VPEs) are found accumulating in ER bodies during biotic stress. These enzymes conduct proteolytic processing of several vacuolar proteins and participate at several stages of plant development and plant cell death. VPEs are classified as cysteine proteases and have properties similar to animal caspases [25]. VPE-containing ER body formation is induced upon biotic stress and eventually, this subgroup of ER bodies fuse with the vacuole [26]. This unique mechanism seems to be highly specific since the trafficking of VPEs has been observed following the classical Golgi-dependent pathway as well [27].

## 3. The Golgi Apparatus and the Trans-Golgi Network/Early Endosome (TGN/EE)

Upon pathogen detection, Golgi-derived vesicles are accumulated at infection sites to facilitate several distinct plant defense responses. Vesicles from the Golgi are involved in fortification of the cell wall by callose [28,29], the secretion of antimicrobial molecules into the apoplast [30] and in transport and regulation of membrane transporters (such as PEN3 and NADPH oxidases) and membrane receptors [31]. 

Prior to fusion, secretory vesicles need to be tethered to the PM by a vesicle tethering complex termed exocyst. This complex comprises eight members (Sec3, Sec5, Sec6, Sec8, Sec10, Sec15, Exo70, and Exo84), and is highly diversified in plants [32]. Two Exo70 members and the Sec5 subunit have been reported to be involved in plant immunity and even described as targets of plant pathogens. Exo70B2 and Exo70H1 are both involved in pathogen responses and mutants displayed increased susceptibility against *Pseudomonas syringae pv. maculicola* [33]. In addition, Exo70B2 has been shown to negatively regulate PAMP-triggered responses and, if perturbed, leads to a higher susceptibility against *Pseudomonas syringae pv* tomato *DC3000* as well [34]. Interestingly, perturbation of Exo70B1 leads to an activated defense status and enhanced resistance against fungal and bacterial pathogens, potentially by interaction with an NLR-like receptor [35]. Recently, it was shown that the *Pseudomonas* effector AvrPtoB associates with and ubiquitinates EXO70B1, stimulating its degradation. This pathogen-induced manipulation impacts on PTI, potentially by inhibiting the secretion of defense-associated molecules [36]. Another exocyst member, Sec5, is required for PR1 secretion and callose deposition as defense measure. In tobacco, it was shown that Sec5 is manipulated by the *Phytophthora infestans* RXLR effector AVR1 facilitating the oomycete infection [37]. Furthermore, inhibition of additional exocyst members, using RNAi lines, also resulted in increased susceptibility to necrotrophic as well as hemibiotrophic pathogens. The finding that the majority of exocyst subunits are involved in callose deposition might explain this general increased susceptibility against various pathogens [38]. Recently, a small molecule, endosidin 2 (ES2), interfering with the activity of a conserved Exo70 subunit was discovered. ES2 affects both animal and plant Exo70 subunits, thereby enhancing vacuolar trafficking and inhibiting secretion. The target of ES2 is the N-terminal part of Exo70A1, which is needed for the membrane association of this subunit [39]. According to this, a rational design approach isolated a structural analog of ES2 (ES2-14) that was more potent against the pathogenic fungi *Botrytis cinerea* and *Magnaporthe. oryzae* than ES2. Although this small molecule has profound effects on growth and exocytosis of growing seedlings, the transient use of this molecule leads to a remarkable reduced lesion formation of both pathogens [40]. 

As part of the exocytosis process, vesicles sorted at the Golgi change their soluble N-ethylmaleimide-sensitive-factor attachment receptor (SNARE) composition and their specific Rab GTPases (“Ras-related in brain”) abundance to bind tethering complexes and finally proceed with its fusion through the activity of tertiary SNARE complexes [41]. In the last decade, research on powdery mildew (*Blumeria graminis)* infection of Arabidopsis yielded different trafficking proteins, among them several SNAREs, involved in non-host resistance responses. Forward as well as reverse genetic approaches with the non-adapted pathogen *B. graminis* revealed the so-called *penetration* (*pen*) mutants 1, 2 and 3 [42,43,44]. These mutants are disabled in non-host penetration resistance and participate in different trafficking pathways. The first protein identified was PEN1, a SNARE at the PM (SYP121) that interacts with the SNAREs SNAP33 and VAMP721/VAMP722 [42]. Together, the tertiary SNARE-complex has been shown to be responsible to mediate resistance to pathogens by callose deposition and the secretion of antimicrobial proteins such as PR1 [45]. PEN2 and PEN3 are involved in a different secretory pathway, which is essential for plant resistance. The myrosinase PEN2 and the ABC-transporter PEN3 mediate callose deposition as well, and furthermore control the production (PEN2) and secretion (PEN3) of Trp-derived antimicrobial glucosinolates [46,47]. Both pathways require rather complex activation mechanisms [48,49] but eventually lead to the rapid relocalization of intracellular PEN1 and PEN3 pools to the PM [31]. As both PEN1 and PEN3 participate in fusion processes at the PM during both abiotic and biotic stresses [49], it is still unclear how exactly pathogens manipulate these pathways. One possibility is that the interaction of the *B. graminis* effector BEC4 with the ARF-GAP AGD5 and an ubiquitin-conjugating enzyme results in degradation of AGD5 [50]. This induced proteasomal degradation could affect the role of AGD5 in the trafficking of PEN1/SYP121 to the PM and other soluble proteins to the vacuole [51,52]. AGD5 interacts with the Golgi-residing GTPase ARF1 and the late Golgi-residing ARF1b/1c, thereby regulating its activity. Notably, an ARF1b/1c mutant has been associated with diminished PEN1 levels at the PM [28]. For PEN3, reorganization of the actin cytoskeleton and Exocyst 84b are necessary for lateral membrane trafficking and polar tethering [53,54]. Interestingly, and in contrast to PEN1, PEN3 is insensitive to the trafficking inhibitor Brefeldin A (BFA) and the pharmacological drug oryzalin, which depolymerizes microtubules. During *B. cinerea* infection, mitogen-activated protein kinases (MAPK3 and MAPK6) are activated and these, in turn, activate the WRKY33 transcription factor. WRKY33 binds the promoter of PEN3 which is responsible for camalexin transport, leading to an enhanced secretion of indole alkaloid phytoalexins [55]. Since PEN1 and PEN3 use different transport routes and have different roles during infection, comprehension of pathogen-induced changes of endomembrane dynamics is the key to deciphering additional plant defense mechanisms thoroughly [48]. For example, in the case of *P.syringae* infection, the already described actin-binding effectors of *P.syringae* are not sufficient to prevent PEN3 relocation to the PM [56,57,58]. On the other hand, upon *B. graminis* attack leading to PEN3 deposition at the PM, disruption of the cytoskeleton results in more rapidly induced autophagic mechanisms that were more effective than PEN3 associated secretion for *B. graminis* resistance [54,59].

For functional analysis of SYP12s related SNAREs, different SNARE mutants were generated and the extracellular proteomes assessed [60]. Overall, pathogen-inducible protein secretion changes were observed in all mutants except *syp42syp43vamp721.* This finding suggests that targeted protein secretion at sites of fungal contact on the leaf epidermal surface depends on the SYP4-VAMP721 secretory pathway. Taken into account that apoplastic fluids from leaves are possibly dominated by proteins secreted from mesophyll cells (not having direct contact with the pathogen), it has been suggested that the SYP4-VAMP721 pathway also contributes to a systemic cell wall remodeling response [60]. In recent years, it became evident that Rab GTPases also plays an important role in plant immunity. Interference of function impairs FLS2 trafficking to and from the cell surface, callose accumulation at infection sites and secretion of antimicrobial proteins (for further reading refer to [61]). These examples underline the necessity of a tight regulation of SNARE and Rab GTPase expression when plants are challenged by biotic and/or abiotic stresses without affecting their constitutive functions [62,63]. 

In addition to the described processes, the NADPH oxidase RBOHD (respiratory burst oxidase homolog protein D) is of eminent importance for plant defense. RBOHD mediates the production of ROS to fight pathogens and is mainly localized to the PM, Golgi cisternae and another, yet unidentified intracellular compartment. Upon pathogen attack, the Golgi pool is rapidly relocated to infection sites at the PM to provide a localized highly oxidative and antimicrobial environment that restricts pathogen growth [64]. ROS production has to be tightly regulated to avoid detrimental effects on host cells. The PM-associated cytoplasmic kinase BIK1 (BOTRYTIS-INDUCED KINASE1) directly interacts with and phosphorylates RBOHD upon PAMP perception. Consequently, inhibition of phosphorylation entirely inhibits RBOHD function within plant immunity [65,66,67]. In wheat, it was shown that in response to pathogen attack, reactive Fe^3+^, but not Fe^2+^accumulated at the cell wall and mediated the oxidative burst [68]. This led to intracellular iron depletion and affected the transcription of pathogenesis-related genes. Interestingly, Fe^3+^ was detected in vesicle-like bodies that were sensitive to actin depolymerization [68]. Since organelle transport depends on actin filaments, it seems plausible that these bodies could be of endosomal nature.

## 4. The Multivesicular Body/Late Endosome

Multivesicular bodies (MVBs)/late endosomes (LEs) have been identified as “prevacuolar” compartments, being en route to the vacuole. Structurally, the MVB/LE is characterized by the possession of intraluminal vesicles (hence the name). Biochemically, MVBs/LEs refers to a vesicle population enriched in the phosphoinositide PI3P (phosphatidylinositol 3-phosphat) and Rab5 GTPases that subsequently matures to a vesicle population enriched in PI (3,5) P_2_ and Rab7 GTPases [69,70]. Besides the transport of vacuolar proteins fulfilling a storage function, MVBs/LEs are responsible for the degradation of ubiquitinated membrane proteins, including receptors from the PM [71,72,73]. Degradation of membrane proteins requires uptake into intraluminal MVB/LE vesicles, which is mediated by the endosomal sorting complex required for transport (ESCRT). Tethering coupled to vesicle fusion and invagination of vesicles performed by the ESCRT complex [74] require the synthesis of PI3P and PI3P-binding proteins that are necessary for the maturation and function of the MVB/LE. The constitutive expression of inactive forms of the ATPase involved in the last step of vesicle internalization leads to a MVB/LE maturation defect similar to that promoted by Rab5 constitutively active (GTP-bound) forms [75,76], confirming that both tethering and vesicle invagination are key steps for MVB/LE function. Recently, the characterization of the unique plant FYVE protein FREE1 has revealed its essential role as an ESCRT member and its interaction with PI3P´s has been demonstrated. RNAi-mediated knockout of FREE1 leads to defects in receptor internalization and perturbed MVB/LE development [77].

Unexpectedly, MVB/LEs have been shown to play a critical role in disease resistance at the cell surface. Many biotrophic fungi penetrate their host and establish feeding structures such as haustoria (e.g., powdery mildew fungi). As a countermeasure, plants redirect trafficking, including MVBs/LEs to penetration sites [42]. Here, so-called papillae are formed in the extracellular space between PM and the cell wall [78]. These papillae reinforce the cell wall by callose deposition and contain antifungal compounds and ROS to efficiently prevent pathogen intrusion [79]. Notably, PEN1/SYP121 was found to localize within papillae and *pen1* mutants showed a delay in formation [80].

Upon pathogen attack, MVBs/LEs fuse with the PM and release their intraluminal vesicles as exosomes (Figure 1B). These might contain antimicrobial compounds, such as phytoalexins, phenolics or ROS (for further reading refer to [81]). Although not entirely understood, this unconventional organelle rerouting seems to be highly important to confer resistance against pathogen entry [79].

Upon PAMP/DAMP recognition, PRRs are internalized from the PM to the TGN/EE. It has been reported that receptor-ligand binding induces clathrin-mediated endocytosis and subsequent transport via the MVB/LE to the vacuole [82]. Here, flg22 is internalized together with the corresponding receptor FLS2 [82,83]. Impaired flg22-FLS2 clathrin-mediated endocytosis results in inhibition of FLS2 associated responses: reactive oxygen species (ROS) production is diminished, callose deposition is impaired and stomatal closure is defective [82]. However, inhibited FLS2 internalization into MVBs/LEs in ESCRT mutants still results in reduced stomata closure, allowing bacterial entry and propagation, but does not inhibit ROS production and MAP kinase cascade activity activation [84]. 

To promote infection, *P. syringae* injects specific effectors, perturbing FLS2 trafficking thereby promoting the establishment of water-soaked lesions leading to higher infection rates [85]. One of these effectors, HopM1, promotes the proteasomal degradation of ARF-GEF MIN7 [86]. MIN7 is involved in BFA-sensitive and actin-dependent recycling of the auxin transporters PINFORMED 1 (PIN1) and PINFORMED 2 (PIN2) [87]. Since constitutive FLS2 trafficking to the MVB/LE has also been shown to be BFA sensitive and dependent on the actin cytoskeleton, perturbing receptor uptake seems to be a relevant strategy of pathogens to suppress PTI. A second known effector, AvrE1 targets the regulatory subunit of the protein phosphatase PP2A that is again involved in FLS2 receptor endocytosis and thereby consequently, in the establishment of FLS2 associated defense responses [88,89]. Furthermore, AvrE1 specifically down-regulates NONRACE-SPECIFIC DISEASE RESISTANCE1/HARPIN-INDUCED1-LIKE13 (NHL13) [90], a host PM protein that contributes to basal plant immunity.

Downstream signaling events such as the activation of pathogen-responsive mitogen-activated protein kinases 3 and 6 (MPK3/6) indirectly contribute to structural MVB/LE changes. MPK3/6 phosphorylate the LYST-INTERACTING PROTEIN 5 (LIP5), which in turn stimulates the ESCRT-associated AAA ATPase SKD1. Notably, disruption of *LIP5* leads to increased susceptibility to the pathogen *P. syringae* while flg22- and salicylic acid-induced defense responses are still largely intact [91]. During infection, an increase of intracellular MVBs/LEs and exosome-like paramural vesicles were observed, but seems to be missorted and accumulate between the PM and the cell wall. It appears likely that the inability of MVB/LE fusion with the vacuole or the PM prevents the delivery of antimicrobial content at the correct location and thus, hinders efficient plant defense responses [91]. 

A similar scenario was shown for the rice SKD1 homolog LRD6-6 [92]. It was demonstrated that LRD6-6 was not only required for MVB/LE-dependent trafficking but also inhibited the biosynthesis of antimicrobial compounds. Disruption of LRD6-6 led to enhanced immunity against *M. oryzae* and in general spontaneous cell death in rice even under non-infected conditions. The authors speculate that this might result from phytoalexin accumulation and might involve impaired CERK1 trafficking which has been shown to regulate immunity [92]. In line with this, *lrd6-6* displays perturbed vacuolar trafficking of the carboxypeptidase Y (CPY) and the adaptor protein complex 3 (AP-3). As the AP-3 complex is involved in endocytosis and has been associated with the acidification of the vacuole [93,94], these results strengthen the hypothesis that perturbed PRR trafficking may be involved in the establishment of the *lrd6-6* phenotype. 

A possible explanation for altered defense signaling observed in trafficking mutants could be related to elevated levels of the phytohormones SA and abscisic acid (ABA). Both hormones do not only inhibit endocytosis [95,96,97] but also impacts on ROS production [98,99] and stomata closure [100,101]. Notably, ROS production itself is involved in the regulation of stomatal movements, which in turn depends on changing vacuolar morphology [102,103]. During ROS production, proline and sugars, functioning as scavengers are released from the vacuole to the cytosol [104]. In addition, ROS production inhibits the glycolytic Glyceraldehyde-3-phosphate dehydrogenase (GPAC1) and recruits it from the cytosol to the MVB/LE. This inhibition leads to an increase of organelle diameter and vacuolar trafficking defects [105,106]. Furthermore, ROS production has been shown to depend on PI3P and diminished levels increase MVB/LE size, vacuolar size and inhibit MVB/LE–vacuole trafficking [107]. Thus, defective pathogen response of MVB/LE mutants seems to be related to elevated phytohormone levels and corresponding defects in ROS production and scavenging. 

Generally, trafficking and subsequent ESCRT-dependent degradation of membrane proteins require post-translational phosphorylation and ubiquitination mechanisms. Impaired ubiquitination has been shown to be important for defense response upon pathogen attack and has been linked to enhanced secretion [108]. A screening for mutants that restore the autoimmunity phenotype of *pen1syp122* led to the isolation of the *amsh3-4pen1syp122* triple mutant, but the cause for this autoimmunity phenotype and the subsequent rescue is not entirely understood [108]. AMSH3 is a deubiquitinating enzyme that promotes the deubiquitination of membrane receptors, and knockout mutants of this protein are seedling lethal [109]. As AMSH3 is needed for (mono) ubiquitination of the chitin receptor CERK and subsequent vacuolar trafficking [110], the reduced catalytic activity of *amsh3-4* in the *pen1syp122* background could compromise CERK associated PTI immunity. 

## 5. The Vacuole

The vacuole, the plant's largest organelle, occupies up to 90% of the cellular volume and fulfills a plethora of different functions. However, one of the less understood functions is the role of the vacuole in plant defense. In general, plant vacuoles accumulate a variety of hydrolytic enzymes and antimicrobial compounds that can be released upon pathogen infection. Despite its importance, the precise composition of accumulated molecules is unknown because proteomic analysis on isolated vacuoles has been carried out under non-infected conditions only [111,112]. Since vacuolar proteases often cover more than one physiological role, it is necessary to decipher their roles in plant immunity individually. Here, vacuole proteomics of infected and neighboring cells (to analyze systemic resistance) at different time points could be a promising strategy. 

**Figure 2:**
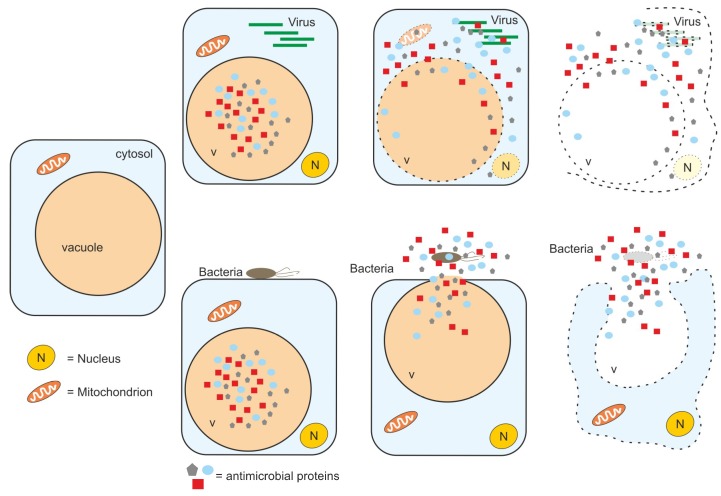
Different release mechanisms of antimicrobial proteins accumulated in the vacuole. The collapse of the vacuolar membrane (tonoplast) upon pathogen invasion (e.g., viruses) leads to plant cell death. Fusion of the tonoplast with the PM leads to the release of antimicrobial proteins to the cell's exterior. This is effective against pathogens proliferating in the apoplast (like some bacteria) and delays cell death.

For the release of antimicrobial proteins at the site of infection, two different mechanisms have been described, both associated with the programmed cell death (PCD) within the hypersensitive response [113]. The first mechanism is initiated upon viral infection and employs VPEs for the disruption of the tonoplast, ultimately leading to the release of the vacuolar contents into the plant cytosol [114]. The second strategy is initiated upon bacterial infection and leads to the fusion of the tonoplast with the PM, leading to the discharge of antibacterial proteases and cell death-promoting contents into the apoplast (Figure 2). 

Since some of these proteins are also expressed under non-infected conditions [115,116,117,118,119], it is difficult to distinguish their roles on cellular homeostasis and plant defense. As pointed out in the ER section, unconventional pathways might be upregulated as part of the developmental program. 

In general, VSRs are responsible for targeting soluble proteins to the vacuole. Pathogen perception involves the expression of soluble vacuolar proteases as part of PTI and ETI. However, these vacuolar proteases are partially secreted, either as a consequence of MVB/LE-membrane fusion or due to VSR transport saturation [120,121]. Although the upregulation of this pathway upon pathogen infection might be possible, vacuolar collapse or fusion with the PM seems more efficient to release antimicrobial proteins. 

Vacuolar collapse results from the expression and activation of vegetative VPEs, during viral and fungal attacks [122,123]. VPEs and other proteases such as RD21 form ER bodies in healthy tissues [26], and are multiplied during infection to fuse with the vacuole. Although the molecular mechanism is not well characterized, the SNARE VAMP714 has been proposed to be a key player for vacuolar collapse during the *M. oryzae* infection in rice [124]. In addition to ER body accumulation and vacuolar collapse, viruses and fungi use the constitutive host endomembrane machinery for replication and establishment of feeding structures such as haustoria. The SNARE VTI11 participates in homotypic vacuole fusion events [125] and was shown to be essential for the replication of the turnip mosaic virus [126]. The resistance mechanism proposed by the authors was that the virus reroutes vesicles in a VTI11-dependent manner in order to reach the extracellular space to spread infection [127]. Endosome-associated VPS9a, a guanine-nucleotide exchange factor for the activation of Rab5 GTPases, on the other hand, is required for both pre- and postinvasive immunity against powdery mildew (*B. graminis*) [48,128].

Under bacterial attack, the fusion of the vacuole with the PM as a release mechanism for antimicrobial proteins is employed [129]. It has been shown that this requires the action of PBA1, a proteasome subunit that is in turn essential for the degradation of ubiquitinated proteins. PBA1 action eventually leads to the activation of caspase 3-like vacuolar processing enzymes and PM-vacuole fusion is induced to promote cell death. In yeast, this mechanism is dependent on Rab7 activity [130]; however, in plants, the exact function of this mechanism remains elusive. In contrast to metazoans, Rab7 has been highly diversified in Arabidopsis, consisting of seven members (RabG3a-g). The specific role of most of the members remains unclear to date. For RABG3b however, positive regulation of autophagy and a role in HR-associated cell death has been described [131,132]. Furthermore, the AP-4 participates in hypersensitive cell death as well, preventing vacuole-PM fusion when mutated [133]. Arabidopsis AP-4 mutants were shown to be susceptible to the avirulent *P. syringae* strain *Pto* DC3000 with the effectors AvrRpm1 or AvrRpt2. Notably, *ap4b-3* and *ap4b-4* showed normal resistance to *Pto* DC3000 with the type III effector AvrRps4. Since *Pto* DC3000 *avrRps4*-induced hypersensitive cell death is extremely weak in wild-type plants, assessment *AP4B* deficiency on this response during RPS4-mediated ETI was not possible [133]. Upon infection with *Pto* DC3000 *avrRpt2*, *ap4b-3* and *ap4b-4* lines did not show vacuole fusion with the PM, whereas the wild-type control did. This indicates that AP-4 participates in cell death-associated immunity, potentially via its contribution to membrane fusion and subsequent release of vacuolar content into the apoplast. 

A striking example of the vacuole´s importance for plant immunity is the existence of so-called myrosin cells (please also refer to the ER-section). These cells are adjacent to the plant's vasculature and accumulate enzymes of the myrosinase family in their vacuoles to catalyze the production of toxic glucosinolate-derivatives. Herbivorous breakage of these cells leads then to the release of these antimicrobial secondary metabolites to prevent further damage. Recently, in addition to the classical myrosinases from family of thioglucoside glucohydrolases (TGGs), the atypical myrosinases PEN2 and PYK10 were identified as key players for the development of myrosin cells [134,135,136]. In contrast to typical myrosinases, PEN2 and PYK10 are not localized in the vacuole but in peroxisomes and in ER bodies. Interestingly, expression of myrosinases was induced when SYP22 and VTI11 function in endosome-vacuole fusion was impaired [137]. 

Apart from vacuolar trafficking, changes in vacuolar morphology contribute to plant immunity as well. This is especially clear, as plants need to actively close their stomata upon contact with microbes to prevent pathogen entry and subsequent colonialization. While closed stomata in guard cells exhibit a fragmented vacuolar pattern at single confocal planes, opened stomata display just one or two large vacuoles, occupying almost the entire cell [138]. Structural organization and dynamics of the vacuole, in turn, rely on the interaction between the actin cytoskeleton and the tonoplast as well as on SNARE proteins [139,140,141]. Notably, the receptor kinase FERONIA integrates both, cell wall-vacuole communication during plant growth [142] and plant immune signaling during pathogen attack [143,144].

## 6. Concluding Remarks

In the last decade, it has been shown that dynamic regulation of the plant endomembrane system is of crucial importance for defense mechanisms beyond PAMP recognition at the PM. To facilitate infection, pathogens actively manipulate secretory, endocytic and vacuolar transport and thus initiate a number of beneficial changes like receptor missorting, muting defense-related signaling and many more. To counteract pathogen-induced manipulation of canonical trafficking routes, plants have developed specific trafficking mechanisms like the formation of ER-bodies and vacuole-PM fusion to fight pathogen attack. These unconventional mechanisms are absent in healthy plants and most of them are not yet well understood. To complicate things, there is also increasing evidence that the fundamental process of autophagy is tightly linked to defense responses against attacking pathogens. Autophagy participates in the regulation of plant cell death, viral clearance, and antimicrobial defenses, but the underlying molecular mechanisms are poorly understood. Notably, pathogens seem to have evolved ways not only to evade autophagic clearance but also to manipulate and subvert autophagy for their own benefit (for further reading refer to [145,146]). 

It will be an exciting task in the future to systematically decipher defense-induced unconventional trafficking pathways in respect to different pathogens. Thereby, common solely defense-related pathways could be the starting point for the generation of robust and more resistant plants against a broad range of pathogens.
